# Relating gas phase to solution conformations: Lessons from disordered proteins

**DOI:** 10.1002/pmic.201400605

**Published:** 2015-06-05

**Authors:** Rebecca Beveridge, Ashley S. Phillips, Laetitia Denbigh, Hassan M. Saleem, Cait E. MacPhee, Perdita E. Barran

**Affiliations:** ^1^ The Michael Barber Centre for Collaborative Mass SpectrometryThe School of Chemistry, Manchester Institute for BiotechnologyUniversity of ManchesterManchesterUK; ^2^ Waters CorporationWilmslowUK; ^3^ School of Physics and AstronomyUniversity of EdinburghEdinburghUK

**Keywords:** Electrospray Ionization Mechanisms, HDX‐MS, Ion Mobility Mass Spectrometry, Parkinson's disease, Technology

## Abstract

In recent years both mass spectrometry (MS) and ion mobility mass spectrometry (IM‐MS) have been developed as techniques with which to study proteins that lack a fixed tertiary structure but may contain regions that form secondary structure elements transiently, namely intrinsically disordered proteins (IDPs). IM‐MS is a suitable method for the study of IDPs which provides an insight to conformations that are present in solution, potentially enabling the analysis of lowly populated structural forms. Here, we describe the IM‐MS data of two IDPs; α‐Synuclein (α‐Syn) which is implicated in Parkinson's disease, and Apolipoprotein C‐II (ApoC‐II) which is involved in cardiovascular diseases. We report an apparent discrepancy in the way that ApoC‐II behaves in the gas phase. While most IDPs, including α‐Syn, present in many charge states and a wide range of rotationally averaged collision cross sections (CCSs), ApoC‐II presents in just four charge states and a very narrow range of CCSs, independent of solution conditions. Here, we compare MS and IM‐MS data of both proteins, and rationalise the differences between the proteins in terms of different ionisation processes which they may adhere to.

## 1 Introduction

The recent years have seen an explosion of research into intrinsically disordered proteins (IDPs) 1. This subset of proteins are flexible and dynamic compared to globular proteins; they populate many interconverting conformations of similar energy and are classified as having no secondary structure on the timescale of an NMR experiment 2, 3, 4. The lack of three‐dimensional structure in IDPs allows them to bind to multiple partners, enabling them to play a key role in many cellular signalling networks 5, 6, 7, 8. It is for this reason that they are frequently implicated in cancers, since a disruption in the function or regulation of IDPs often results in a breakdown of cell division control leading to uncontrolled cell proliferation.

It is convenient to consider that IDPs have similar characteristics to structured proteins that have been denatured by the solution conditions, but this is an oversimplification. The dynamic properties of IDPs under physiological conditions differ from those of denatured structured proteins due to differences in their hydrodynamic behaviour. Rather than behaving as random coils, IDPs are often relatively compact compared to denatured globular proteins; transient elements of secondary structure reduce the hydrodynamic radius, giving rise to conformations of differing compactness 1, 9. The extent of this compactness differs between IDPs due to differing levels of intramolecular non‐covalent interactions such as hydrogen bonds and electrostatic interactions, as well as being affected by the solution conditions 10.

The lack of fixed structure in IDPs causes challenges when gathering structural information; they do not readily crystallise and NMR is unable to provide information on interconverting populations. While disorder can be detected by NMR spectroscopy by the chemical shifts of disordered residues, it is unable to report on more specific residual information since several conformations are interconverting on a timescale that is faster than that over which the NMR experiment takes place. Electrospray ionisation–mass spectrometry (ESI–MS) has provided valuable information on dynamic ensembles of IDPs; it is sensitive to the degree of disorder and can represent the full conformational range of an IDP 11. In ESI‐MS, proteins are observed in a range of charge states which, in positive ionisation mode, are commonly due to protonated forms of the protein. Structured proteins have a limited number of solvent‐accessible ionisable sites and hence display in a narrow range of charge states upon ionisation 12. Disordered proteins, however, exist in a range of conformations, from compact to extended, with differing numbers of protonatable sites at the surface of the proteins. IDPs, therefore, display a wide charge state range which reflects their dynamic behaviour and allows distinction between different conformations of the same protein 13, 14.

Ion mobility (IM) is a gas phase electrophoretic technique which can be used to give an extra experimental dimension to MS data. During an ion mobility experiment, a packet of ions is pulsed into a drift tube, across which is applied a weak electric field. The ions are drawn though the cell, but are hindered by collisions with an inert buffer gas (in this case helium) which is at a known temperature and pressure. The velocity of a given ion is influenced by two factors: the shape and the charge, both of which will determine the number of collisions with the buffer gas and how quickly the ion is pulled through the drift cell [15]. In a typical IM–MS experiment we record the drift time of m/z separated species and from this measurement we can calculate the buffer gas dependent rotationally averaged collision cross section (Ω) which correlates to the available conformation(s) of any given protein 16. IM–MS has a particular application in the study of IDPs 17, 18, 19, 20.

The mobility of an ion is determined as the ratio of the drift velocity (*v*_d_) and applied electric field (E). It is then possible to determine the CCS on the basis of Eq. (1):

(1) $$K_{0}=\frac{3ze}{16N}{\left(\frac{2\pi }{\mu k_{B}T}\right)}^{1/2}\frac{1}{\Omega },$$

where *z* is the ion charge state, *e* is the elementary charge, *N* is the gas number density, μis the reduced mass of the ion‐neutral pair, *k_B_* is the Boltzman constant, *T* is the gas temperature and *K*_0_ is the reduced mobility (the measured mobility *K* standardised for pressure and temperature to 273.15 K and 760 Torr).

**Figure 1:**
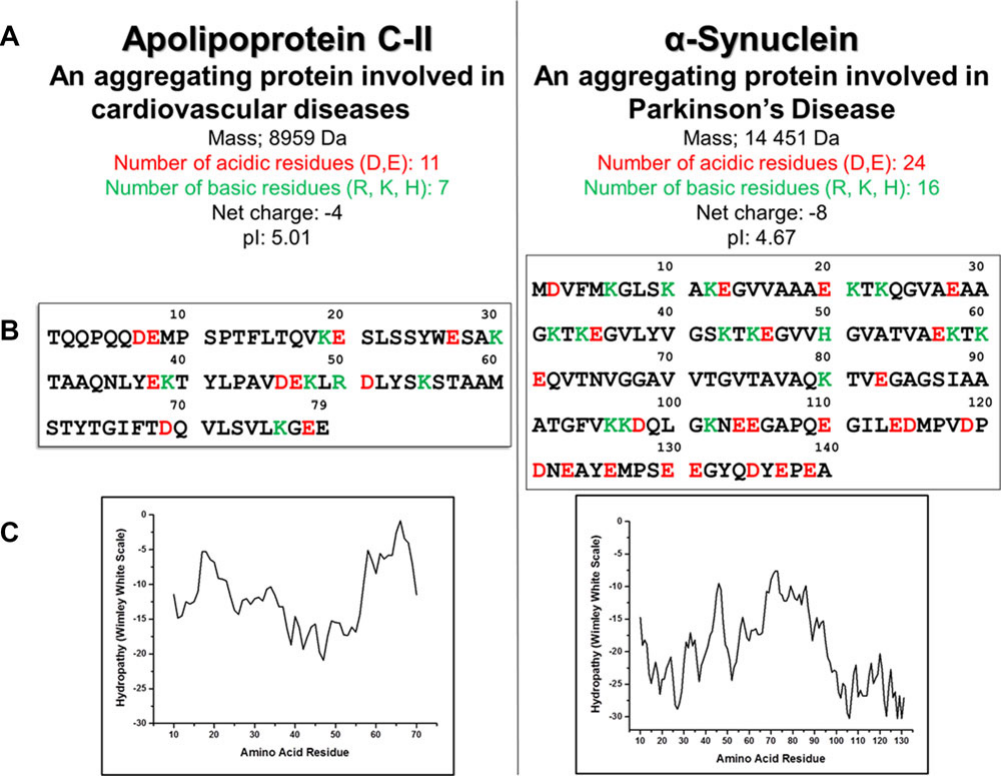
Comparison of the biophysical characteristics of ApoC‐II and α‐Synuclein. (a) General properties of the proteins. (b) Sequences showing positive and negative residues in green and red, respectively. (c) Wimley–White plots showing the hydrophobicity of the proteins.

Hydrogen–deuterium exchange coupled with mass spectrometry (HDX–MS) is a sensitive and rapid technique with which to investigate both structure and dynamics of proteins in solution. HDX‐MS can localise protected areas within proteins; that is areas which are not accessible to the solvent due to secondary or tertiary structure 21. During a typical HDX‐MS workflow, the protein of interest is diluted into deuterated buffer, and the exchange of solvent deuterium atoms is allowed to proceed for a given amount of time. The reaction is then quenched by the addition of a low pH buffer which reduces the back‐exchange of deuterated backbone amides. The protein is digested by an acid‐stable protease, in this case pepsin, and the HDX extent is analysed by MS of the protein fragments. Because IDPs contain few protected amide hydrogens due to their lack of tertiary structure, peptides from such proteins are expected to be fully saturated with deuterium at the earliest on‐exchange time point 22. Here, we use HDX‐MS and ESI‐IMS‐MS to study two proteins which have been shown by many techniques to be intrinsically disordered; α‐Syn and ApoC‐II (Fig. 1).

α‐Syn is a protein of 140 amino acids (14 460 Da) which is highly expressed in the brain and is the primary component of the Lewy body deposits found in dopaminergic neurons that characterise Parkinson's disease (PD) and other neurodegenerative diseases. Despite being the focus of much research, its exact function is still unknown, but it is perhaps one of the most thoroughly investigated IDPs due to its role in PD. While α‐Syn tends to be natively disordered, it has been shown that alterations to the solution environment, either an increase in hydrophobicity or a decrease in pH, can induce partial folding 23. ApoC‐II is a plasma protein containing 79 amino acids (8959 Da) and is involved in lipid transport and metabolism. ApoC‐II is a protein activator of lipoprotein lipase which is an enzyme that hydrolyses triacylglycerol during the metabolism of chylomicrons and very low‐density lipoproteins 24.

Both ApoC‐II and α‐Syn are classified as being IDPs 25, 26. They both have a net negative charge; ‐4 and ‐8 for ApoC‐II and α‐Syn, respectively, and no hydrophobic regions. The Wimley–White hydrophobicity scale is a combination of two different scales; one which considers the enthalpy change upon transfer of unfolded domains from water into the lipid bilayer, and one which considers the enthalpy change upon transfer of folded chains into the hydrocarbon interior. These two scales are then combined to create a whole residue hydropathy plot which accounts not only for the side chains, but also the peptide bonds, both of which are important to consider 27. Because neither of the proteins examined here have any regions which would preferably exist in a hydrophobic environment than an aqueous one, it can be assumed that hydrophobicity for both the proteins is very low; the more positive the value, the more hydrophobic are the amino acids in that segment of the protein. Here, all the values for both the proteins are negative (figure 1C), indicating a predominantly hydrophilic amino acid composition. It is worth noting that this scale assesses the likelihood of a protein segment forming a transmembrane helix and does not account for binding to the surface of membranes, which is discussed below.

ApoC‐II and α‐Syn have both been investigated in the presence of lipids. ApoC‐II plays a role in plasma lipid metabolism; it binds reversibly to the polar lipid surface of plasma lipoprotein particles in vivo and also to a range of synthetic and natural lipid surfaces in vitro 28 with a corresponding change in the secondary structure characteristics. MacRaild et al. have demonstrated the propensity of ApoC‐II to fold into α‐helices both in the presence of sodium dodecyl sulphate 29 and dodecyl phosphocholine 30. It has also been shown that α‐Syn forms helices in the presence of lipids 31, 32. Moreover, it has been documented that both proteins form amphipathic α‐helices when bound to a synthetic membrane. When the residues of α‐Syn are plotted onto a helical wheel 33, several regions have polar and non‐polar residues distributed on opposite sides of the helix 31, agreeing very closely to amphipathic α‐helix found in the lipid binding domains of the exchangeable lipoproteins, a family of which ApoC‐II is a member.

As described above, the biophysical characteristics of ApoC‐II and α‐Synuclein are similar. They have similar primary structures in terms of lots of charged residues, net negative charge and low hydrophobicity. They have a similar pH and they both bind to lipid membranes in a similar fashion which includes the formation of amphipathic α‐helices. Because of all these similarities it could be hypothesised that the two proteins under scrutiny will display in a similar fashion in the gas phase. However, we have found major differences in the presentation of these two proteins to the gas phase from solution, as shown by MS and IM–MS data which we attribute to differences in the electrospray ionisation mechanism. This is supported by the findings from HDX–MS which reports on the solvated structure of a given protein, and both proteins are indicated to have a conformation which is completely solvent accessible even on a short timescale (15s).

## 2 Materials and methods

### 2.1 Sample preparation

α‐Synuclein was expressed recombinantly, from a pT7‐7 vector containing human α‐Synuclein gene, kindly provided by Professor Chris Rochet, Purdue University and purified as described previously 34; a Resource Q column (GE Healthcare Life Sciences, UK) was used. α‐Synuclein was concentrated using Vivaspin 6, MWCO 10 kDa centrifugal sample concentrators (GE Healthcare Life Sciences, UK) and applied to a HiPrep 26/10 desalting column (GE Healthcare Life Sciences, UK), pre‐equilibrated with 100 mM ammonium acetate (Fisher Scientific, UK), flow rate 10 mL/min. The eluent was lyophilised and stored at –80°C. Prior to use, α‐Synuclein was resuspended in 50 mM ammonium acetate. ApoC‐II was bacterially expressed and purified as previously described 35. The pET11a/human ApoC‐II construct was kindly provided by Associate Professor Geoff Howlett, University of Melbourne. The purified protein was buffer exchanged into the relevant MS‐compatible solutions using Bio‐Rad micro Bio‐Spin P‐6 Columns.

### 2.2 Hydrogen–deuterium exchange

Protein solutions were prepared of 6 μM ApoC‐II and 20 μM α‐Syn. HDX labelling and quenching procedures were automatically performed using the CTC PAL sample manager (LEAP Technologies, Carrboro, NC, USA). The samples were diluted with 10 mM phosphate in 99.99% deuterium oxide, pH 6.6 (pD 7.0) 30‐fold and 20‐fold for ApoC‐II and α‐Syn, respectively, and incubated for 0, 15 and 60 s at 20°C. Labelled samples were then quenched with an equal volume of pre‐chilled 100 mM phosphate pH 2.5. All labelling time‐points were analysed in triplicate. 50 μL of sample was injected on a nanoACQUITY UPLC™ system with HDX technology (Waters). Online pepsin digestion for ApoC‐II was performed in 0.1% formic acid for 2 min at 20°C on a Poroszyme immobilised pepsin cartridge (Applied biosystems). For α‐Syn, this was performed for 1 min at 20˚C on a Waters Enzymate^TM^ immobilised BEH pepsin column (2.1 × 30 mm). The peptides were separated on a UPLC BEH C18 column (Waters) at 0°C. ApoC‐II peptides were separated with a 6 min linear acetonitrile gradient (8–40%) containing 0.1% formic acid at 40 μL/min. α‐Syn peptides were separated with a 7 min linear acetonitrile gradient (8–35%) containing 0.1% formic acid at 40 μL/min. Mass spectra were acquired on a SYNAPT G2‐Si HDMS in MS^E^ mode over the m/z range of 50–2000. Non‐deuterated peptides were identified using ProteinLynx Global Server software 3.1 (Waters). DynamX 2.0 software (Waters) was used to filter the peptides, to generate deuterium uptake plot for each of them and to visualise the deuteration on the protein sequence.

### 2.3 Mass spectrometry and ion mobility

Nano‐electrospray ionisation (nESI) was used for all MS and IM–MS experiments. Solutions were ionised through a positive potential applied to a thin platinum wire inserted into a thin‐walled glass capillary (inner diameter 0.9 mm, outer diameter 1.2 mm, World Precision Instruments, Stevenage, UK) that was pulled to an nESI tip in house with a Flaming/Brown micropipette puller (Sutter Instrument Co., Novato, CA).

### 2.4 Mass spectrometry

MS experiments were performed on a Q‐ToF Ultima (Waters, Manchester, UK). α‐Syn samples (concentration 70 μM) were sprayed from solutions of 50 mM ammonium acetate pH 6.8 or pH 3.5. ApoC‐II samples (concentration 30 μM) were sprayed from solutions of 10 mM ammonium acetate pH 6.8, 100 mM ammonium acetate pH 6.8, 100 mM ammonium acetate pH 2.5 or 100% MeOH, as outlined in the text/Fig. 4. Capillary voltage 1.6–1.9 kV, cone voltage 60–100 V, source temperature 80°C, collision energy 5.

### 2.5 Ion mobility–mass spectrometry

IM–MS experiments were carried out on a Waters Q‐ToF I instrument that was modified in house to include a 5.1 cm drift tube which has been described elsewhere 15. The temperature and pressure of helium in the drift cell were approximately 28˚C and 3.7 Torr, respectively. Measurements were made at six different drift voltages from 60 to 20 V. The precise pressure and temperature were recorded for every drift voltage and used in the calculations of CCSs. Each experiment was performed in triplicate. Data were analysed using MassLynx v4.1 software (Waters, Manchester, UK), Origin v8.5 (OriginLab Corporation, USA) and Microsoft Excel. Ion arrival time distributions were recorded by synchronisation of the release of ions into the drift cell with mass spectral acquisition. The CCS distribution plots are derived from raw arrival time data using Eq. (2) 36.

(2) Ω avg =18π1/2161mb+1m1/2zeKBT1/21ρtdVL2,

where *m* and *m_b_* are the masses of the ion and buffer gas, respectively; *z* is the ion charge state; *e* is the elementary charge; *K_B_* is the Boltzmann constant; *T* is the gas temperature; ρ is the buffer gas density; *L* is the drift tube length; *V* is the voltage across the drift tube and *t_d_* is the drift time.

The raw arrival time output (*t_a_*) includes time the ions spend outside of the drift cell but within the mass spectrometer, known as the dead time (*t_0_*). The value for *t_0_* is calculated by taking an average value of the intercept from a linear plot of average arrival time versus pressure/temperature and was subtracted from the arrival time to calculate drift time 15 (*t_D_*):

(3) $$t_{D}=t_{a}-t_{0}.$$

### 2.6 Wimley–White plot

Hydrophobicity scales were calculated using the MPEX software found at http://blanco.biomol.uci.edu/mpex/ 27. Sequences can be found in Fig. 1B.

### 2.7 Modelling of theoretical CCS extremities

This procedure has been described elsewhere 37. Briefly, the lower boundary was calculated by assuming that the globular form of the protein approximates a spherical shape with a density of ρ = 0.904 Da/Å^3^. Using the molecular weight *M_w_* of the protein, the volume of the protein sphere can be calculated via *V* = *M_w_*/ρ.The radius of the sphere is, therefore, *r* = (3*V*/4π)^1/3^. The collision cross section of a sphere of this radius is given by Eq. (1).

(4) CC S lower Å2=πr2=π3V4π2/3.

The upper boundary can be calculated by assuming that the protein is cylindrical in shape; the furthest distance between α‐carbons in a protein chain is 3.63 Å 32. Therefore for a polypeptide of *n* residues, the maximum linear dimension is *n*(3.63) Å 32. The radius is given by the geometric average of the sum of the radii of the amino acids contained in the protein's sequence. The average volume of an amino acid in a protein's sequence is given by Eq. (2):

(5) V¯=∑i amino  acids ViNin.

Here, the sum is over all amino acids *i*, *V_i_* is the volume of the *i*th amino acid, *N_i_* is the number of amino acids of type *i* in the protein sequence. The average amino acid radius is then approximated by *r* = (*V*/π*h*)^1/2^, where *h* (height) = 3.63 Å. This forms the radius of the fully extended protein cylinder. The collision cross section is then given by the rotationally averaged collision cross section of this cylinder since the protein ‘tumbles’ in the drift tube of the apparatus due to the low electric field.

The rotationally averaged collision cross section is given by the projection area of the cylinder

(6) CC S upper Å2= Projection  area  of  cylinder =4πrl+2r2.

These values are then multiplied by a scaling factor of 1.19 to convert from geometric size to CCS in helium as outlined in 37. These theoretical values are highly approximate and do not take into consideration disulphide bridges, proline residues or other non‐covalent interactions or restrictions, but serve as upper and lower boundaries with which to compare experimental data.

## 3 Results and discussion

### 3.1 The use of HDX–MS to examine the solvent accessibility of the proteins α‐Syn and ApoC‐II

**Figure 2:**
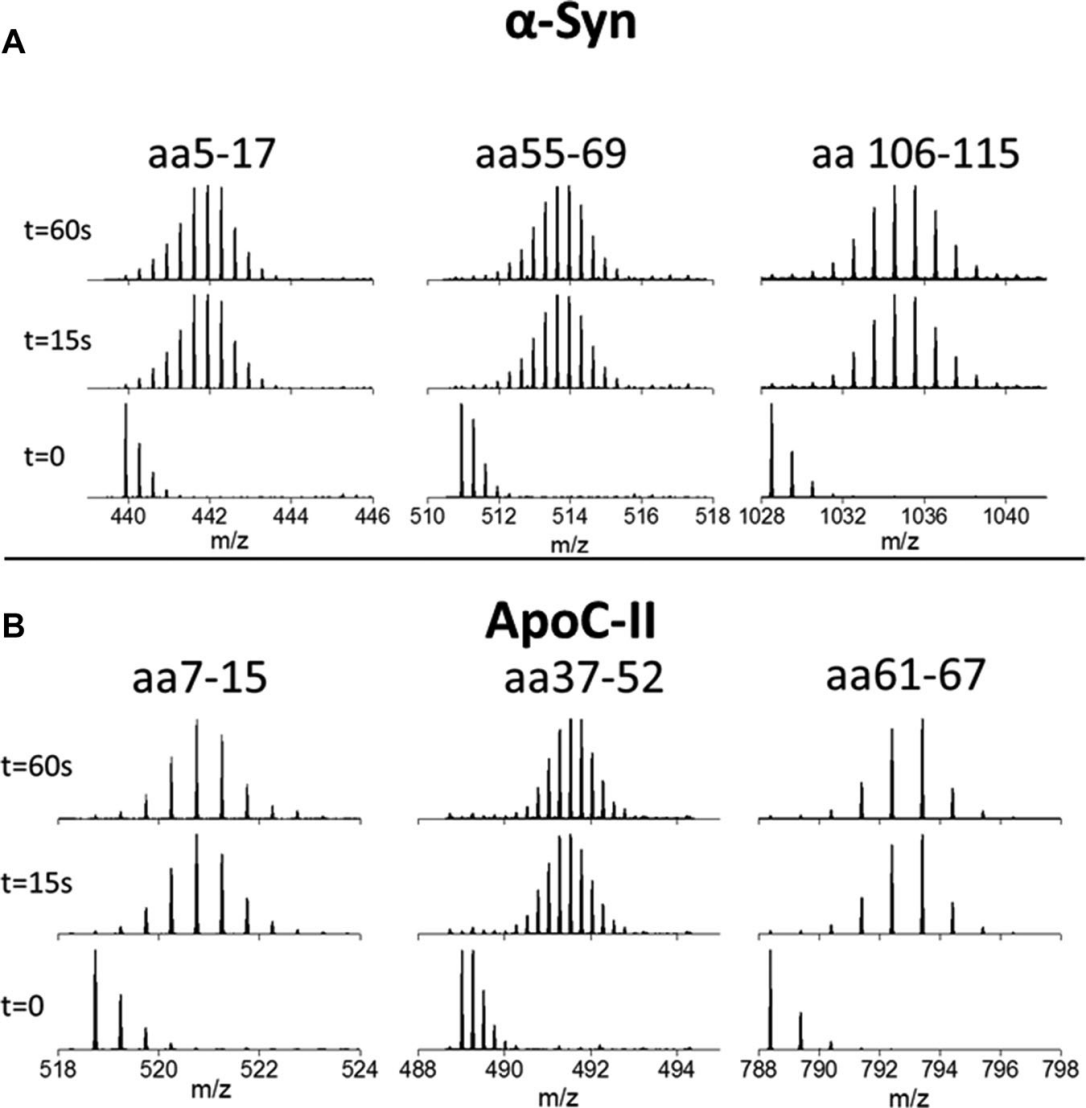
HDX data of three peptides of α‐Syn (top) and ApoC‐II (bottom). For each of the three peptides, the mass change at 15 s indicates that the maximum number of type II deuteriums that could exchange for hydrogens, have already been exchanged. For further details, see SI Table 3)

HDX–MS was used to confirm previous results that α‐Syn and ApoC‐II behave in a similar fashion in solution; as disordered proteins with little secondary structure. Such proteins are expected to undergo hydrogen–deuterium exchange rapidly since there is no protection of the backbone amide protons from the solvent due to the adoption of structure by the protein. This is indeed what is observed for both proteins. Figure 2 illustrates this, it shows MS data over a time course of exposure to a deuterated solution for three peptides from each protein. We have selected one from each terminal region and one roughly in the middle, but these are typical for all peptides found from each protein. Three time points are shown for each peptide; *t* = 0 (non‐deuterated), *t* = 15 s and *t* = 60 s. In each case, no more deuteration is observed at *t* = 60 s than *t* = 15 s. This demonstrates that at just 15 s, all the deuteration that is possible has already occurred and there has been no prevention by secondary structure of the protein. This confirms that both proteins are flexible and dynamic under these solution conditions.

### 3.2 The analysis of charge state distributions to probe the accessibility of chargeable sites in α‐Syn and ApoC‐II following nESI–MS

**Figure 3:**
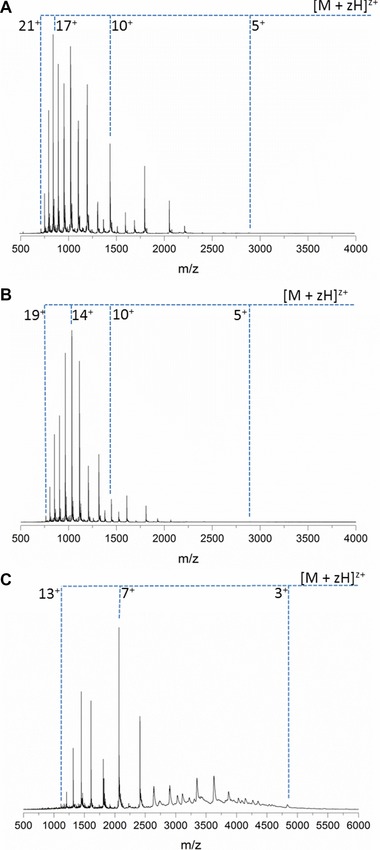
nESI of α‐Syn. In both (A) and (B), the solution conditions are identical (50 mM ammonium acetate pH 6.8) but the CSD differs. In (C) the ionic strength has been increased to 100 mM ammonium acetate and the pH lowered to 3.5. The measured mass of the protein is 14 453 Da *cf*. the theoretical average mass, 14 460 Da.

nESI of α‐Syn from 50 mM ammonium acetate (Fig. 3A) produces ions ranging in charge state from [M+5H]^5+^ to [M+20H]^20+^. This wide charge state distribution (CSD) is very typical of IDPs 38. Because of the lack of secondary structure the protein is free to adopt many conformations, ranging from compact to extended, hindered only by weak energetic constraints. This is reflected in the CSD of the protein. The compact conformations contribute the lower charge states seen since there are few ionisable sites that are accessible to the solvent for protonation. As conformations become more extended there are more solvent accessible protonable sites which gain charges and contribute the higher charge states.

α‐Syn produces a very variable CSD, as seen in figures A and B, which were taken under the exact same conditions and instrumental parameters. The fact that the CSD displayed by α‐Syn is so variable further highlights the plasticity of the protein. The folding landscapes of structured proteins have one energy minima in which all or most of the molecules reside. This is characterised by a narrow CSD of five or fewer charge states. Some IDPs, for example β‐casein, have structured regions that are joined together by regions of disorder allowing transitions between several low energy minima 39. This is displayed by a wide CSD of more than five charge states, but with higher intensity peaks for the lower charge states 38. Even most IDPs will have a shallow well in the folding landscape which represents a preferential conformation; proteins with a wide CSD often have a particular profile which is reproducible. The fact that α‐Syn has such a wide CSD with variation in the most intense peaks suggests that the folding landscape is shallow and there are very few energetic constraints preventing access to certain shapes. This information from ESI–MS characterisation is in agreement with the HDX data shown in Fig. 2.

When the pH is reduced to pH 3.5 there is a compaction of the protein conformation (Fig. 3C). The most intense charge state is [M+7H]^7+^ which is lower than in either of the spectra taken at pH 6.8. This compaction has been observed by other biophysical techniques and is frequent in IDPs 40.

**Figure 4:**
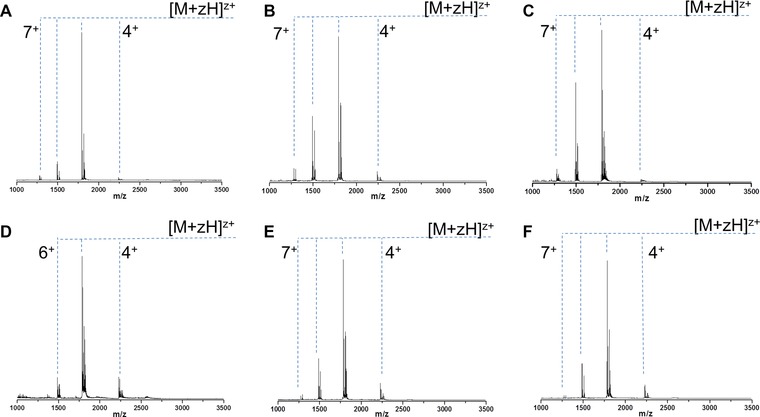
Mass spectra of ApoC‐II taken under different solution conditions (A–D) and sprayed from different temperatures (E, F). (a) 10 mM ammonium acetate pH 6.8. (B) 100 mM ammonium acetate pH 6.8. (C) 100 mM ammonium acetate pH 2.5. (d) Methanol, (E) 100 mM ammonium acetate, 80˚C, (F) 100 mM ammonium acetate, 14˚C. Measured mass = 8 959 Da.

nESI of ApoC‐II from 10 mM ammonium acetate produces monomers ranging in charge state from [M+4H]^4+^ to [M+7H]^7+^, with most of the ions in the [M+5H]^5+^ charge state (figure 4A). This narrow CSD is unusual for an IDP, and contrasts greatly with the signature shown by α‐Syn in Fig. 1. Increasing the ionic strength, by raising the concentration of ammonium acetate to 100 mM, often causes a compaction of the protein conformation and a resulting shift to lower charge states; here it has very little effect on the CSD (Fig. 4A and B). Although reducing the pH of the solution conditions tends to have a denaturing effect on structured proteins, for IDPs there is little secondary structure to disrupt so the results are often minimal. Here, although there is a slight increase in the intensity of the [M+6H]^6+^ charge state, the overall CSD is unchanged and remains narrow (Fig. 4C). Adding a hydrophobic solvent often has the same denaturing effect as low pH, and it is highly unusual that spraying the protein from 100% methanol produces a narrower CSD than buffered conditions; the [M+7H]^7+^ ion is totally depleted (Fig. 4D). After showing that the CSD cannot be altered by changing the solvent environment, it was investigated whether physical changes could induce any effect. The temperature of the capillary from which the protein solution was sprayed was altered, and spectra were recorded at 14 and at 80˚C (Fig. 4E and F). Even altering the temperature by such a large extent produces no change in the CSD. Although HDX experiments suggest an extended conformation of ApoC‐II in solution, MS experiments indicate a compact conformation in the gas phase, regardless of solution conditions or temperature.

### 3.3 The use of IM‐MS to probe the conformational spread of the gas phase forms of α‐Syn and ApoC‐II

**Figure 5:**
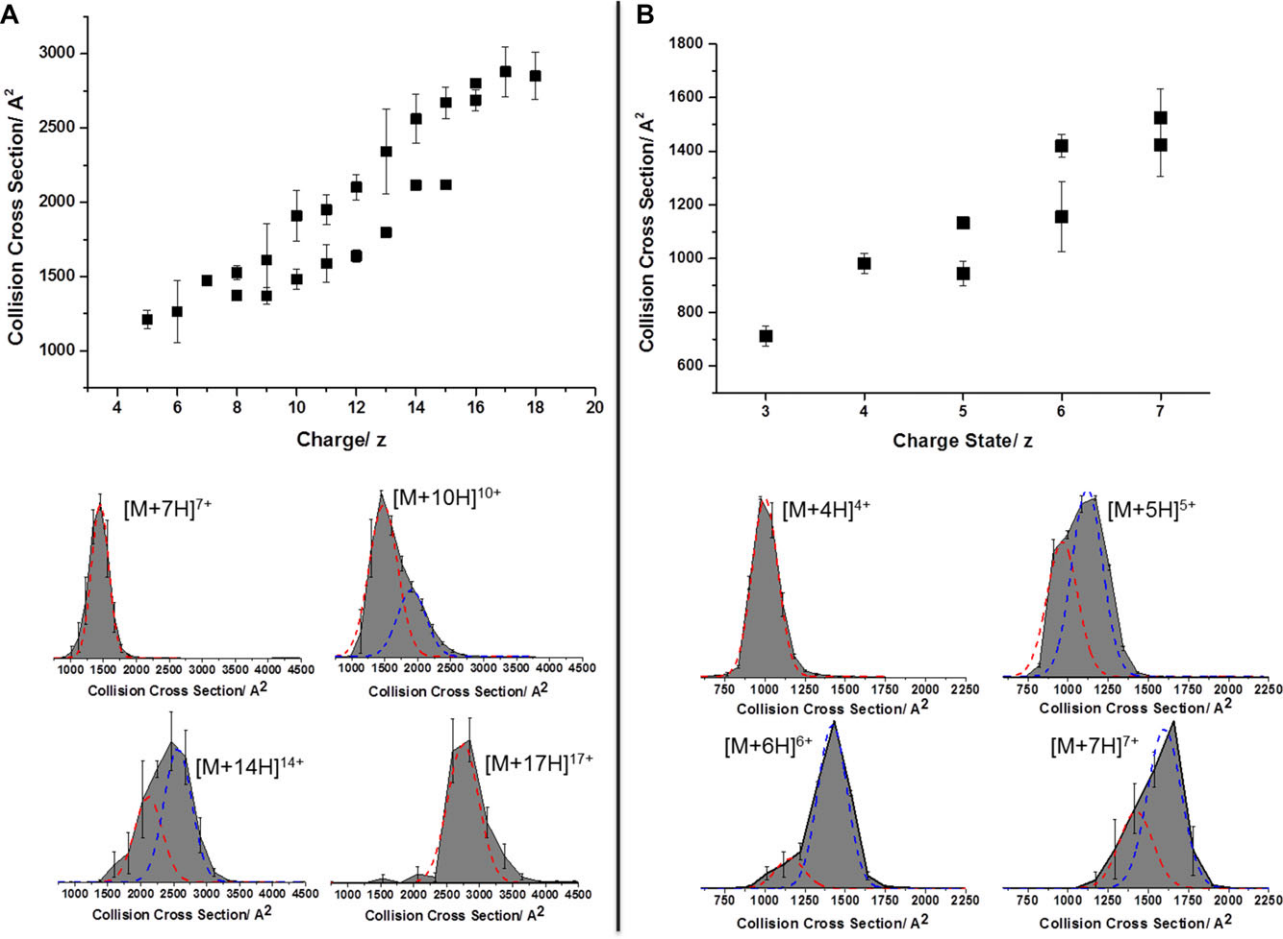
Ion mobility data of α‐Synuclein (A) and ApoC‐II (B). Collision cross sections of each charge state (top) and arrival time distributions of selected charge states (bottom).

IM analysis provides additional and complementary information to the MS results shown earlier. Rotationally averaged collision cross sections have been measured for each charge state (Fig. 5 and Table S1 and S2 in the supporting information). The apex of the collision cross section distributions found for α‐Syn range from 1043 Å^2^ for the [M+5H]^5+^ ion to 2742 Å^2^ for the [M+18H]^18+^ ion. Two distinct conformational families are observed for the ions carrying between 8 and 16 positive charges, with just one conformational family for the lower and higher charge states. The increase in collision cross section with respect to charge begins to level off at the [M+16H]^16+^, presumably as the addition of extra charges cannot induce any further coulombic unfolding as the protein is already in an almost fully extended state. This is typical behaviour of an IDP and can be observed on other systems 38.

The collision cross sections measured for ApoC‐II are starkly different from that of α‐Syn. For ApoC‐II, the CCSs range from 945 Å^2^ for the smaller conformation of the [M+5H]^5+^ ion to 1524 Å^2^ for [M+7H]^7+^. Apart from [M+4H]^4+^ all charge states are present in two conformational families, with the more extended conformational family being more intense.

There is a wealth of information in the width of the collision cross section distributions of the proteins. The widths of the ApoC‐II ATDs are ∼700, 750, 900 and 950 Å^2^ for the increasing charge states. For α‐Syn, the widths are 1250, 1850, 2100 and 1700 Å^2^ for [M+7H]^7+^, [M+10H]^10+^, [M+14H]^14+^ and [M+17H]^17+^, respectively. There is much more variance of CCS in each charge state of α‐Syn. This could either be due to the presence of multiple conformers which we are unable to resolve, or interconversion of conformations on the timescale of the experiment. One way to discern which is occurring would be to carry out the experiments under reduced temperatures to see if it is possible to freeze out specific conformers. Either way, the wider ATD for the α‐Syn charge states indicates more conformational disparity.

### 3.4 The use of a simple model to predict conformational occupancy

We have developed a simple approach to predict the CCS of the most compact and most extended forms of both proteins, as described above in the method section and in 38. A comparison of measured CCS values with those predicted for any protein sequence reveals how much of the possible conformational space that *might* be occupied by a given protein *actually is*. Using this model, we have previously demonstrated 38 that proteins that are known to be structured under given solution conditions present with a narrow range of CCS compared to that which they could occupy. By contrast, disordered proteins or proteins that are sprayed from denaturing conditions give an experimental ΔCCS that covers much of the predicted allowed space. Using this approach, α‐Syn behaves as predicted for an IDP. The extremities of CCSs are predicted to be 743 Å^2^ and 3380 Å^2^ (ΔCCS 2637 Å^2^) for the smallest and the largest, respectively. The smallest measured CCS is 870 Å^2^ and the largest is 3249 Å^2^. The collision cross section ranges are therefore 2637 and 2379 Å^2^ for the theoretical and measured values, respectively. This means that the calculated:measured CCS range ratio of α‐Syn is 0.9, or rather that 90% of the allowed space is occupied

For ApoC‐II the calculated collision cross section range is 873–2786 Å^2^ (ΔCCS = 1913 Å^2^) while the measured range is 945–1524 Å^2^ (ΔCCS = 579 Å^2^). This means that in contrast to a‐Syn, ApoC‐II has a calculated:measured CCS range ratio of just 0.3. In addition, the largest conformation is just 55% of the size of the largest possible cross section that was calculated, and this is due to the low intensity [M+7H]^7+^ ion; most of the ions are in the [M+5H]^5+^ charge state which provides two conformational families of just 945 and 1132 Å^2^, the most intense of which is the larger. According to the mass spectrum, 86% of ions are present at [M+5H]^5+^. The ΔCCS of this charge state alone is 187 Å^2^, which gives a calculated:measured CCS range of 0.10. The calculated:measured CCS range ratio for cytochrome c is 0.13, for lysozyme is 0.24 and myoglobin is 0.19. These are all proteins with a very specific three‐dimensional structure that is required for their function. The occupancy for ApoC‐II is not very different from these structured proteins when the full CSD is considered and is much smaller when percentage occupancy is taken into account, even though it is thought to exist in a dynamic ensemble of conformations in solution.

Although ApoC‐II is 5 kDa smaller than α‐Syn this does not account for the differences in observed gas phase conformations. A similar sized protein to ApoC‐II is ubiquitin which has been widely investigated in MS and IM–MS studies. Ubiquitin sprayed from denaturing conditions has been reported to have a Δ*z* = 8 and a ΔCCs = 998 41, with a calculated:measured CCS range of 0.62; much larger than that of ApoC‐II. This is the conformational spread which can be expected in IM–MS experiments of a disordered form of ApoC‐II.

### 3.5 Discussion and implications for the nESI process for these proteins

**Figure 6:**
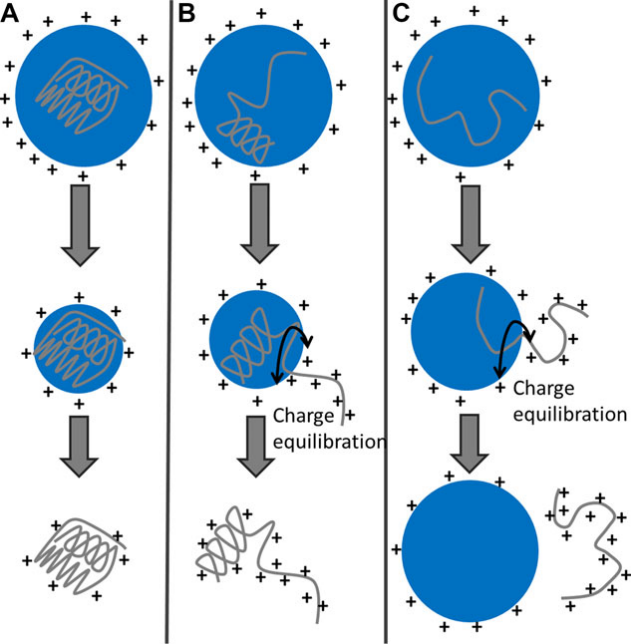
Three proposed electrospray mechanisms; (A) and (C) (the charged residue model and the chain ejection model, respectively) were proposed by Konermann et al. 44. We also proposed an intermediate between the two previously described mechanisms (B). Figure adapted from Ref. [44].

The variance in the recorded CSDs and the collision cross section distributions for these two examples of protein that in solution are conformationally dynamic, raises interesting questions regarding what happens as they desolvate. Figure 6 shows three possible electrospray processes that a protein undergoing desolvation can adhere to. Figure 6A shows the charge residue mechanism (CRM) which is a well‐established mechanism for the desolvation of large and relatively lowly charged macromolecular species which are ‘structured’ [43, 44] and 6c shows the chain ejection mechanism (CEM), recently proposed by Konermann et al. 44. During the CRM process, it has been hypothesised that Rayleigh‐charged nanodroplets containing a single analyte evaporate to dryness. They lose charge as the droplet shrinks, via the ejection of protons and small ions, which allows the droplet to remain close to the Rayleigh limit 37 as the size decreases. Remaining protons are transferred to the protein during the final stages of desolvation.

Konermann et al. 44 have used MD simulations to demonstrate that unfolded and unstructured proteins are transferred from the droplet into the gas phase via a CEM. This is attributed to their more extended nature, with exposed hydrophobic groups making it more difficult for the protein to reside in the droplet interior. Such proteins therefore readily migrate to the surface. One terminus gets expelled into the vapour phase, followed by stepwise ejection of the remaining protein and separation from the droplet. The prevailing view is that the CRM is adhered to when proteins are present in solution in compact, and/or globular forms, whereas extended conformations will undergo a CEM. Therefore, structured proteins with a fixed tertiary conformation will commonly follow the CRM to produce ions with a median charge that is low, and satisfies the Rayleigh relationship 37.

Disordered proteins are capable of existing in a range of conformations, ranging from compact to extended, unhindered by energetic constraints. They also contain forms where a given region is compact and another is extended. These three types of conformation must all be accounted for in the transfer of protein to the gas phase. If, in the nanodroplet, an IDP happens to be in a compact conformation, it enters the gas phase via the CRM, resulting in the lower charge state region of the mass spectrum. The higher charges will be contributed by proteins undergoing the CEM which will take place if the protein in the nanodroplet is in an extended conformation. This leaves the intermediate charge states to rationalise a mechanism for. Are they comprised of ions that may have desolvated via either (or both) of the above mechanisms? To answer this we propose an intermediate mechanism which will be followed by conformations with regions that are compact and regions that are extended. During this mechanism the extended part of the protein will be ejected from the droplet as in the CEM, but this will be an unfavourable way to remove compact region of the protein from the droplet which is more likely to undergo the CRM. The differing amount of the polypeptide which is in an extended or compact state will therefore give rise to intermediate charge states, and also multiple conformations within the same charge state.

The wide CSD of α‐Syn demonstrates that it has multiple conformations in solution which give rise to a wide CSD due to different conformations undergoing different ionisation mechanisms. Some of these conformations will be capable of binding to membranes, however the mechanism for this remains elusive. Because ApoC‐II is also disordered in solution, we hypothesise that a wide CSD would be observed for this proteins also, however as described in this paper this was not the case. A narrow CSD centred on low charge states was observed from all tested solution conditions. This implies that conformational switching occurs during the very last stages of desolvation to provide a compact form that always presents to the gas phase as having undergone a CRM.

Ogorzalek Loo et al. have recently also described an intermediate region in the electrospray process where the desolvating ions have properties lying between those of solution‐phase and gas‐phase 45. They contact the solvent transiently in a high electric field, facilitating proton redistribution to confer the most stability to the emerging gas‐phase ion. We speculate that extended forms of gas‐phase ApoC‐II are extremely unfavourable during this intermediate phase, resulting in charge redistribution and conformational switching to a compact, favourable conformation. This may be because it is a membrane interacting protein, stabilised in a lower solution dielectric. A similar effect was previously reported from IM–MS and HDX–MS studies of melittin 46, which was seen to rearrange to a helical structure upon desolvation, irrespective of solution conformation.

## 4 Concluding remarks

Here, we have analysed two proteins by several MS methods; HDX–MS, nESI–MS and nESI‐IM–MS. The proteins, namely α‐Syn and ApoC‐II, have both previously been assigned as IDPs and share many biophysical characteristics, as outlined in Fig. 1. Firstly, the proteins were analysed via HDX–MS which confirmed that both proteins are disordered in solution, in agreement with previous studies 26, 35. We then analysed the proteins via nESI–MS which shows stark differences in terms of CSD. α‐Syn, as expected for an IDP, displays a very wide range of charges in fitting with a lack of structure 38. Disparity is observed between different spectra taken under the same conditions, further highlighting the plasticity of the protein. ApoC‐II however is present in no more than four charge states, irrelevant of the solution conditions from which it is sprayed, indicating a small number of gas‐phase conformations. IM–MS experiments support the nESI–MS experiments of both proteins. A wide range of CCSs are measured for α‐Syn. CCS tends to increase with charge, and there are also multiple conformations within each charge state. ApoC‐II is present in a narrow range of CCSs, but also has more than one conformational family for most charge states. Comparison with a theoretical model which predicts the CCSs of the most extended and most compact conformations of a protein shows that α‐Syn explores most of the conformational space that is available to it, whereas ApoC‐II is only present in a small amount of available conformational space. Finally, we rationalise the range of conformations displayed in the gas phase by each protein by the ESI mechanisms that they both adhere to. Our research into disordered systems is forcing us to think more deeply about the processes that occur during the transfer of proteins to the gas phase, and teaching us more about electrospray.

*The authors have declared no conflict of interest*.

## Supporting information

As a service to our authors and readers, this journal provides supporting information supplied by the authors. Such materials are peer reviewed and may be re‐organized for online delivery, but are not copy‐edited or typeset. Technical support issues arising from supporting information (other than missing files) should be addressed to the authors.

Table S1. Rotationally averaged collision cross sections of ApoC‐II
Table S2. Collision cross sections of 50μM α‐synuclein in 50mM ammonium acetate
Table S3. Deuterium uptake of ApoC‐II and α‐Syn fragments. The theoretical maximum uptake is *n*‐1, where *n* is the number of amino acids in the peptide [1].

## References

[1] Uversky, V. N. , Dunker, A. K. , Understanding protein non‐folding. Biochim. Biophys. Acta (BBA) ‐ Proteins & Proteomics 2010, 1804, 1231–1264. 20117254 10.1016/j.bbapap.2010.01.017 PMC2882790

[2] Ward, J. J. , Sodhi, J. S. , McGuffin, L. J. , Buxton, B. F. et al., Prediction and functional analysis of native disorder in proteins from the three kingdoms of life. J. Mol. Biol. 2004, 337, 635–645. 15019783 10.1016/j.jmb.2004.02.002

[3] Daughdrill, G. W. , Hanely, L. J. , Dahlquist, F. W. , The c‐terminal half of the anti‐sigma factor FlgM contains a dynamic equilibrium solution structure favoring helical conformations. Biochemistry 1998, 37, 1076–1082. 9454599 10.1021/bi971952t

[4] Tompa, P. , Unstructural biology coming of age. Curr. Opin. Struct. Biol. 2011, 21, 419–425. 21514142 10.1016/j.sbi.2011.03.012

[5] Dunker, A. K. , Cortese, M. S. , Romero, P. , Iakoucheva, L. M. et al., Flexible nets ‐ the roles of intrinsic disorder in protein interaction networks. Febs J. 2005, 272, 5129–5148. 16218947 10.1111/j.1742-4658.2005.04948.x

[6] Uversky, V. N. , Oldfield, C. J. , Dunker, A. K. , Showing your ID: intrinsic disorder as an ID for recognition, regulation and cell signaling. J. Mol. Recognit. 2005, 18, 343–384. 16094605 10.1002/jmr.747

[7] Singh, G. P. , Dash, D. , Intrinsic disorder in yeast transcriptional regulatory network. Proteins‐Struct. Funct. Bioinform. 2007, 68, 602–605. 10.1002/prot.21497 17510967

[8] Xie, H. B. , Vucetic, S. , Iakoucheva, L. M. , Oldfield, C. J. et al., Functional anthology of intrinsic disorder. 1. Biological processes and functions of proteins with long disordered regions. J. Proteome Res. 2007, 6, 1882–1898. 17391014 10.1021/pr060392u PMC2543138

[9] Dunker, A. K. , Lawson, J. D. , Brown, C. J. , Williams, R. M. et al., Intrinsically disordered protein. J. Mol. Graph. Model. 2001, 19, 26–59. 11381529 10.1016/s1093-3263(00)00138-8

[10] Mueller‐Spaeth, S. , Soranno, A. , Hirschfeld, V. , Hofmann, H. et al., Charge interactions can dominate the dimensions of intrinsically disordered proteins. Proc. Natl. Acad. Sci. USA 2010, 107, 14609–14614. 20639465 10.1073/pnas.1001743107 PMC2930438

[11] Natalello, A. , Benetti, F. , Doglia, S. M. , Legname, G. et al., Compact conformations of alpha‐synuclein induced by alcohols and copper. Proteins‐Struct. Funct. Bioinform. 2011, 79, 611–621. 10.1002/prot.22909 21120859

[12] Konermann, L. , A minimalist model for exploring conformational effects on the electrospray charge state distribution of proteins. J. Phys. Chem. B 2007, 111, 6534–6543. 17511498 10.1021/jp070720t

[13] Frimpong, A. K. , Abzatimov, R. R. , Uversky, V. N. , Kaltashov, I. A. , Characterization of intrinsically disordered proteins with electrospray ionization mass spectrometry: conformational heterogeneity of alpha‐synuclein. Proteins‐Struct. Funct. Bioinform. 2010, 78, 714–722. 10.1002/prot.22604 PMC5815318 19847913

[14] Konermann, L. , Collings, B. A. , Douglas, D. J. , Cytochrome c folding kinetics studied by time‐resolved electrospray ionization mass spectrometry. Biochemistry 1997, 36, 5554–5559. 9154939 10.1021/bi970046d

[15] McCullough, B. J. , Kalapothakis, J. , Eastwood, H. , Kemper, P. et al., Development of an ion mobility quadrupole time of flight mass spectrometer. Anal. Chem. 2008, 80, 6336–6344. 18627133 10.1021/ac800651b

[16] Bohrer, B. C. , Merenbloom, S. I. , Koeniger, S. L. , Hilderbrand, A. E. et al., Biomolecule analysis by ion mobility spectrometry. Annu. Rev. Anal. Chem. 2008, 1, 293–327. 10.1146/annurev.anchem.1.031207.113001 PMC3780392 20636082

[17] Maurizio, E. , Cravello, L. , Brady, L. , Spolaore, B. et al., Conformational role for the C‐terminal tail of the intrinsically disordered high mobility group A (HMGA) chromatin factors. J. Proteome Res. 2011, 10, 3283–3291. 21545188 10.1021/pr200116w

[18] Brocca, S. , Testa, L. , Sobott, F. , Samalikova, M. et al., Compaction properties of an intrinsically disordered protein: Sic1 and its kinase‐inhibitor domain. Biophys. J. 2011, 100, 2243–2252. 21539793 10.1016/j.bpj.2011.02.055 PMC3149264

[19] Canon, F. , Ballivian, R. , Chirot, F. , Antoine, R. et al., Folding of a salivary intrinsically disordered protein upon binding to tannins. J. Am. Chem. Soc. 2011, 133, 7847–7852. 21524106 10.1021/ja200534f

[20] Faull, P. A. , Korkeila, K. E. , Kalapothakis, J. M. , Gray, A. et al., Gas‐phase metalloprotein complexes interrogated by ion mobility‐mass spectrometry. Int. J. Mass Spectrom. 2009, 283, 140–148.

[21] Katta, V. , Chait, B. T. , Hydrogen/deuterium exchange electrospray ionization mass spectrometry: a method for probing protein conformational changes in solution. J. Am. Chem. Soc. 1993, 115, 6317–6321.

[22] Goswami, D. , Devarakonda, S. , Chalmers, M. , Pascal, B. et al., Time Window Expansion for HDX analysis of an intrinsically disordered protein. J. Am. Soc. Mass Spectrom. 2013, 24, 1584–1592. 23884631 10.1007/s13361-013-0669-y PMC3773365

[23] Uversky, V. N. , Li, J. , Fink, A. L. , Evidence for a partially folded intermediate in α‐synuclein fibril formation. J. Biol. Chem. 2001, 276, 10737–10744. 11152691 10.1074/jbc.M010907200

[24] Havel, R. J. , Fielding, C. J. , Olivecrona, T. , Shore, V. G. et al., Cofactor activity of protein components of human very low density lipoproteins in the hydrolysis of triglycerides by lipoprotein lipase from different sources. Biochemistry 1973, 12, 1828–1833. 4349259 10.1021/bi00733a026

[25] Tajima, S. , Yokoyama, S. , Kawai, Y. , Yamamoto, A. , Behavior of apolipoprotein C‐II in an aqueous solution. J. Biochem. 1982, 91, 1273–1279. 7096287 10.1093/oxfordjournals.jbchem.a133812

[26] Uversky, V. N. , A protein‐chameleon: conformational plasticity of α‐synuclein, a disordered protein involved in neurodegenerative disorders. J. Biomol. Struct. Dyn. 2003, 21, 211–234. 12956606 10.1080/07391102.2003.10506918

[27] Snider, C. , Jayasinghe, S. , Hristova, K. , White, S. H. , MPEx: a tool for exploring membrane proteins. Protein Sci. 2009, 18, 2624–2628. 19785006 10.1002/pro.256 PMC2821280

[28] Hanson, C. L. , Ilag, L. L. , Malo, J. , Hatters, D. M. et al., Phospholipid complexation and association with apolipoprotein C‐Ii: insights from mass spectrometry. Biophy. J. 2003 85, 3802–3812. 10.1016/S0006-3495(03)74795-X PMC1303682 14645070

[29] MacRaild, C. A. , Hatters, D. M. , Howlett, G. J. , Gooley, P. R. , NMR structure of human apolipoprotein C‐II in the presence of sodium dodecyl sulfate†. Biochemistry 2001, 40, 5414–5421. 11331005 10.1021/bi002821m

[30] MacRaild, C. A. , Howlett, G. J. , Gooley, P. R. , The structure and interactions of human apolipoprotein C‐II in dodecyl phosphocholine†,‡. Biochemistry 2004, 43, 8084–8093. 15209504 10.1021/bi049817l

[31] Davidson, W. S. , Jonas, A. , Clayton, D. F. , George, J. M. , Stabilization of α‐synuclein secondary structure upon binding to synthetic membranes. J. Biol. Chem. 1998, 273, 9443–9449. 9545270 10.1074/jbc.273.16.9443

[32] Eliezer, D. , Kutluay, E. , Bussell Jr, R. , Browne, G. , Conformational properties of α‐synuclein in its free and lipid‐associated states. J. Mol. Biol. 2001, 307, 1061–1073. 11286556 10.1006/jmbi.2001.4538

[33] Schiffer, M. , Edmundson, A. B. , Use of helical wheels to represent the structures of proteins and to identify segments with helical potential. Biophys. J. 1967, 7, 121–135. 6048867 10.1016/S0006-3495(67)86579-2 PMC1368002

[34] Wood, S. J. , Wypych, J. , Steavenson, S. , Louis, J. C. et al., Alpha‐synuclein fibrillogenesis is nucleation‐dependent ‐ implications for the pathogenesis of Parkinson's disease. J. Biol. Chem. 1999, 274, 19509–19512. 10391881 10.1074/jbc.274.28.19509

[35] Hatters, D. M. , MacPhee, C. E. , Lawrence, L. J. , Sawyer, W. H. et al., Human apolipoprotein C‐II forms twisted amyloid ribbons and closed loops†. Biochemistry 2000, 39, 8276–8283. 10889036 10.1021/bi000002w

[36] Jarrold, M. F. , Unfolding, refolding, and hydration of proteins in the gas phase. Acc. Chem. Res. 1998, 32, 360–367.

[37] Fernandez de la Mora, J. ., Electrospray ionization of large multiply charged species proceeds via Dole's charged residue mechanism. Anal. Chim. Acta 2000, 406, 93–104.

[38] Beveridge, R. , Covill, S. , Pacholarz, K. J. , Kalapothakis, J. M. D. et al., A mass‐spectrometry‐based framework to define the extent of disorder in proteins. Anal. Chem. 2014, 86, 10979–10991. 25353392 10.1021/ac5027435

[39] Livney, Y. D. , Schwan, A. L. , Dalgleish, D. G. , A study of β‐casein tertiary structure by intramolecular crosslinking and mass spectrometry. J. Dairy Sci. 2004, 87, 3638–3647. 15483147 10.3168/jds.S0022-0302(04)73502-X

[40] Tompa, P. , Fersht, A. , Structure and function of intrinsically disordered proteins, CRC Press, Boca Raton, FL 2010.

[41] Valentine, S. J. , Counterman, A. E. , Clemmer, D. E. , Conformer‐dependent proton‐transfer reactions of ubiquitin ions. J. Am. Soc. Mass Spectrom. 1997, 8, 954–961.

[42] Kebarle, P. , Verkerk, U. H. , Electrospray: from ions in solution to ions in the gas phase, what we know now. Mass Spectrom. Rev. 2009, 28, 898–917. 19551695 10.1002/mas.20247

[43] Iavarone, A. T. , Williams, E. R. , Mechanism of charging and supercharging molecules in electrospray ionization. J. Am. Chem. Soc. 2003, 125, 2319–2327. 12590562 10.1021/ja021202t PMC1343448

[44] Konermann, L. , Ahadi, E. , Rodriguez, A. D. , Vahidi, S. , Unraveling the mechanism of electrospray ionization. Anal. Chem. 2013, 85, 2–9. 23134552 10.1021/ac302789c

[45] Ogorzalek Loo, R. , Lakshmanan, R. , Loo, J. , What protein charging (and supercharging) reveal about the mechanism of electrospray ionization. J. Am. Soc. Mass Spectrom. 2014, 25, 1675–1693. 25135609 10.1007/s13361-014-0965-1 PMC4163133

[46] Florance, H. V. , Stopford, A. P. , Kalapothakis, J. M. , McCullough, B. J. et al., Evidence for [small alpha]‐helices in the gas phase: a case study using melittin from honey bee venom. Analyst 2011, 136, 3446–3452. 21701716 10.1039/c1an15291b

