# Supplementary material for: Relating gas phase to solution conformations: Lessons from disordered proteins
Source: Proteomics. 2015 Jun 5;15(16):2872–83. doi: 10.1002/pmic.201400605 (PMC4744708; doi:10.1002/pmic.201400605)
Supplement: Supplementary file 1 — Table S1. Rotationally averaged collision cross sections of ApoC‐II Table S2. Collision cross sections of 50μM α‐synuclein in 50mM ammonium acetate Table S3. Deuterium uptake of ApoC‐II and α‐Syn fragments. The theoretical maximum uptake is n‐1, where n is the number of amino acids in the peptide [1]. [file PMIC-15-2872-s001.docx]

| Charge State | Rotationally Averaged Collision Cross Section/ Å^2^ | Standard Deviation |
| --- | --- | --- |
| [M+4H]^4+^ | 982 | 38 |
| [M+5H]^5+^ | 945 | 46 |
| [M+5H]^5+^ | 1132 | 13 |
| [M+6H]^6+^ | 1156 | 130 |
| [M+6H]^6+^ | 1420 | 43 |
| [M+7H]^7+^ | 1424 | 119 |
| [M+7H]^7+^ | 1524 | 107 |

Table 1 Rotationally averaged collision cross sections of ApoC-II.

| Species | m/z | Average collision cross section (no. of repeats)/ Å^2^ | Standard deviation |
| --- | --- | --- | --- |
| [M+5H]^5+^ | 2893 | 1043 (2) | 133 |
| [M+6H]^6+^ | 2411 | 1217 (3) | 147 |
| [M+7H]^7+^ | 2067 | 1252 (3) | 147 |
| [M+8H]^8+^ | 1809 | 1333 (3) | 181 |
| [M+9H]^9+^ | 1608 | 1506 (3) | 248 |
| [M+10H]^10+^ | 1447 | 1951 (3) | 155 |
| [M+11H]^11+^ | 1316 | 2161 (3) | 173 |
| [M+12H]^12+^ | 1206 | 2311 (3) | 243 |
| [M+13H]^13+^ | 1113 | 2150 (3) | 398 |
| [M+14H]^14+^ | 1034 | 2446 (3) | 136 |
| [M+15H]^15+^ | 965 | 2476 (3) | 161 |
| [M+16H]^16+^ | 904 | 2560 (3) | 229 |
| [M+17H]^17+^ | 851 | 2617 (3) | 153 |
| [M+18H]^18+^ | 804 | 2742 (2) | 11 |
| [M+19H]^19+^ | 762 | 2605 (3) | 183 |
| [M+20H]^20+^ | 724 | 2620 (1) | - |

Table 2 Collision cross sections of 50µM α-synuclein in 50mM ammonium acetate

| Peptide | Theoretical maximum deuterium uptake | Mass increase at apex of isotopic distribution / Da | Maximum mass increase/ Da |
| --- | --- | --- | --- |
| α-Syn residues 5-17 | 11 | 5 | 10 |
| α-Syn residues 55-69 | 14 | 9 | 14 |
| α-Syn residues 106-115 | 9 | 6 | 9 |
| ApoC-II residues 7-15 | 8 | 4 | 8 |
| ApoC-II residues 37-52 | 15 | 8 | 15 |
| ApoC-II residues 61-67 | 6 | 5 | 6 |

Table 3 Deuterium uptake of ApoC-II and α-Syn fragments. The theoretical maximum uptake is n-1, where n is the number of amino acids in the peptide [[1](#_ENREF_1)].

1. Noble, A.J., Q. Zhang, J. O'Donnell, H. Hariri, et al., *A pseudoatomic model of the COPII cage obtained from cryo-electron microscopy and mass spectrometry.* Nat Struct Mol Biol, 2013. **20**(2): p. 167-173.
